# Induction of cotton ovule culture fibre branching by co-expression of cotton *BTL*, cotton *SIM*, and *Arabidopsis STI* genes

**DOI:** 10.1093/jxb/ert222

**Published:** 2013-08-21

**Authors:** Gaskin Wang, Hongjie Feng, Junling Sun, Xiongming Du

**Affiliations:** State Key Laboratory of Cotton Biology, Institute of Cotton Research of Chinese Academy of Agricultural Sciences, Anyang 455000, Henan Province, China

**Keywords:** Branch, endoreduplication, fuzz, *Gossypium*, lint, nuclear DNA content.

## Abstract

The highly elongated single-celled cotton fibre consists of lint and fuzz, similar to the *Arabidopsis* trichome. Endoreduplication is an important determinant in *Arabidopsis* trichome initiation and morphogenesis. Fibre development is also controlled by functional homologues of *Arabidopsis* trichome patterning genes, although fibre cells do not have a branched shape like trichomes. The identification and characterization of the homologues of 10 key *Arabidopsis* trichome branching genes in *Gossypium arboreum* are reported here. Nuclear ploidy of fibres was determined, and gene function in cotton callus and fibre cells was investigated. The results revealed that the nuclear DNA content was constant in fuzz, whereas a limited and reversible change occurred in lint after initiation. *Gossypeum arboreum BRANCHLESS TRICHOMES* (*GaBLT*) was not transcribed in fibres. The homologue of *STICHEL* (*STI*), which is essential for trichome branching, was a pseudogene in *Gossypium*. Targeted expression of *GaBLT*, *Arabidopsis STI*, and the cytokinesis-repressing *GaSIAMESE* in *G. hirsutum* fibre cells cultured *in vitro* resulted in branching. The findings suggest that the distinctive developmental mechanism of cotton fibres does not depend on endoreduplication. This important component may be a relic function that can be activated in fibre cells.

## Introduction

Although the majority of plant trichomes are multicellular, those of *Arabidopsis* trichomes (leaf hairs) and *Gossypium* (cotton)—unique among crop plants—consist of extremely elongated single cells. The four commercially domesticated species of cotton (*Gossypium hirsutum*, *G. herbaceum*, *G. arboreum*, and *G. barbadense*) produce two types of seed fibres (lint and fuzz) in distinct waves. Lint production is usually initiated before or on the day of anthesis, whereas the development of fuzz fibres from rapidly enlarging spherical cells occurs in a second wave a few days later.

*Arabidopsis* trichomes are rarely formed adjacent to each other, and fate determination relies on a substrate depletion and lateral inhibition mechanism (Ishida *et al.*, 2008; Pesch and Hulskamp, 2009). The activators of trichome fate in *Arabidopsis* are *TRANSPARENT TESTAGLABRA1* (*TTG1*) and *GLABRA1*, *2*, and *3* (*GL1*, *GL2*, and *GL3*). In cotton, the R2R3 MYB genes *GhMYB109* and *GaMYB2*, which have high sequence similarities to *GL1* and *GaHOX1* (homologous to *GL2*), are specifically expressed in the early stages of fibre cell development. Ectopic expression of these cotton genes under control of the *GL1* or *GL2* promoter can restore formation of trichomes in their corresponding mutants (Suo *et al.*, 2003; Wang *et al.*, 2004). Functional homologues of *Arabidopsis TTG1* and *GL3* have also been cloned from *G. hirsutum* (Humphries *et al.*, 2005; Shangguan *et al.*, 2008). Anatomic and morphological observations reveal that cotton fibres seldom form clustering, like that observed for *Arabidopsis* trichomes, suggesting that a similar mechanism is involved in the regulation of fibre spacing. These findings suggest that the *Arabidopsis* genes involved in trichome formation pathways may also participate in cotton fibre patterning processes (Serna and Martin, 2006).

Upon *Arabidopsis* trichome initiation, a unique branched cellular architecture immediately appears, with mature *Arabidopsis* leaf trichomes typically having three branches borne on a stalk. Cells destined to become protrichomes exit the mitotic stage and enter into the endoreduplication progress. Protrichome cells form branches and then expand during endoreduplication. Endoreduplication is a modified cell cycle in which nuclear chromosomal DNA is repeatedly replicated without subsequent cell division (Kasili *et al.*, 2011). *Arabidopsis* SIAMESE (SIM), a plant-specific cell cycle regulator, controls the onset of trichome endoreduplication (Churchman *et al.*, 2006), and BRANCHLESS TRICHOMES (BLT) regulates endoreduplication levels (Bramsiepe *et al.*, 2010; Kasili *et al.*, 2011). Aside from the endoreduplication pathway, genetic and molecular data indicate that nine different loci influence branching via independent molecular pathways. *KATANIN1* (*KTN1*), *SPIKE1* (*SPK1*), *ZWICHEL* (*ZWI*), *ETHYLENE RECEPTOR2* (*ETR2*), *KIESEL* (*KIS*), and *PORCINO* (*POR*) participate in microtubule biogenesis (Oppenheimer *et al.*, 1997; Burk *et al.*, 2001; G.T. Kim *et al.*, 2002; Kirik *et al.*, 2002; Qiu *et al.*, 2002; Plett *et al.*, 2009). *ANGUSTIFOLIA* (*AN*) localizes to punctate structures around the Golgi apparatus (Minamisawa *et al.*, 2011) and is thought to regulate Golgi-related processes (Hülskamp, 2004), whereas *STOMATAL CYTOKINESIS-DEFECTIVE 1* (*SCD1*) regulates cytokinesis (Falbel *et al.*, 2003; Kang *et al.*, 2003). In addition to these genes, *STICHEL* (*STI*) initiates trichome branch formation in a dose-dependent manner via an unknown mechanism (Ilgenfritz *et al.*, 2003; Kasili *et al.*, 2011). With the exception of *sim*, mutations in each of the above genes lead to trichome hypobranching. If cotton fibre cells are homologous to trichomes, then why are cotton fibres unbranched, and are they capable of branching? In this study, both of these long-standing questions are assessed.

Here, it is shown that endoreduplication was not concomitant with fibre outgrowth, corresponding to the *BTL* homologue being epistatic in fibre. Fibres could be induced to branch using targeted gene co-expression of (i) cotton *BTL* to elevate endoreduplication levels; (ii) *Arabidopsis STI* to replace the pseudogene in cotton; and (iii) cotton *SIM* to promote the mitosis to endoreduplication transition in developing fibre cells.

## Materials and methods

### Plant materials and growing conditions

*Gossypium arboreum* cv. DPL971, its natural fuzzless mutant DPL972, and *G. hirsutum* cv. MD51 ne were grown in field nurseries of a greenhouse at 25–32 °C with ~14h illumination. Bolls were selected from the same boll-bearing branches. Flowers were dated on the day they opened. This day was defined as 0 day post-anthesis (0 DPA). Roots, stems, and leaves were collected 20 d after germination, unless otherwise noted.

### Genome DNA extraction and Southern blots

General procedures for DNA manipulation were as described by Sambrook and Russell (2001). Southern blots were performed under both low- and high-stringency conditions. Stringency is defined as the degree of complementarity. Depending on evolutionary distance, gene sequences in cotton and *Arabidopsis* may have diverged extensively. Low-stringency hybridization permitted the probe to bind to related but not necessarily identical genes in the same family or to locate a gene in *G. arboreum* that was similar to a known gene in *Arabidopsis*. With high-stringency hybridization, the probe ideally bound to a completely complementary sequence. Digoxigenin-labelled high-stringency hybridization was performed in accordance with the Cheunglab public protocol (http://www.dartmouth.edu/~staphy/protocol/southern_with_dig_probe.html). Low-stringency hybridization was carried out at 42 °C, and the membranes were washed with 0.2× SSC (0.3M sodium chloride, 0.03M sodium citrate, pH 7.0) and 0.1% (w/v) SDS.

### RNA analysis

Total RNA was isolated using Column Plant RNAout 2.0 (Tiandz, Beijing, China). For RNA *in situ* hybridization, cotton ovules were collected and fixed in a solution of 4% (w/v) formaldehyde and 0.1% (v/v) Tween-20 in diethylpyrocarbonate-treated phosphate-buffered saline (PBS). The fixed ovules were processed according to the protocol described by Mayer *et al.* (1998). The samples were infiltrated with paraffin. Tissue sections were 8 µm in thickness. Digoxigenin-11 UTP (Roche) was incorporated into antisense or sense RNA probes. Preparation of radiolabelled probes and northern blot analysis were carried out as described by Sambrook and Russell (2001). Equal mixtures of 3, 5, 7, 10, 15, 20, and 25 DPA ovule or fibre RNA were used.

### Data analysis

The whole-genome uncombined shotgun sequence of the wild diploid cotton species *G. raimondii*, which was sequenced and made available by Monsanto and Illumina, was downloaded from the GenBank database. A local Blast search was carried out using the related genes of *Arabidopsis* as the queries. Sequence information was extracted if the *E*-value was ≤10.

### Nuclear isolation and DNA content measurement

Procedures for nuclear isolation and DNA microspectrophotometric measurements (using a CRAIC microspectrophotometer) of 20 and 25 DPA fibre cells were as previously described (Van’t Hof, 1999). The nuclear DNA content of *G. arboreum* fibre specimens was estimated by comparing fluorescence values with those of chicken erythrocytes, based on 2C DNA=2.4 pg as the standard (Clowes *et al.*, 1983; Kapraun and Nguyen, 1994). *Gossypium arboreum* root tip cells were used as the 2C reference value standard. DPL971 root tips (3–4cm in length) were harvested and fixed in formaldehyde as described above. After rinsing in phosphate buffer for 10min, cell walls were enzymatically degraded by incubating root tips in 2.0% (w/v) cellulase R-10 (Sigma) and 0.5% (w/v) pectinase (Sigma) for 1.5h at 37 °C in a rotary shaker at 30rpm. Nuclear isolation and DNA measurements were performed as described above.

For determining early-stage ovule DNA content, the ovule fixation, subsequent sucrose infiltrations, embedding, sectioning at 50 µm, and dehydration were as previously described (Wu *et al.*, 2007). The MMI CellCut laser microdissection system (Molecular Machines & Industries) was used for laser capture of epidermal cells. Cell wall enzymolysis, nuclear isolation, and DNA measurements of the collected cells were performed as described above.

For flow cytometric analysis, *in vitro* cultured calli were chopped with a razor blade in 0.1M citric acid and 0.5% (v/v) Tween-20, and the nuclear suspension was prepared using the two-step protocol described by Doležel *et al*. (2007). The nuclei were analysed using the BD FACSVerse flow cytometer.

### Protein–protein interaction assays and western blot analysis

Entry clones containing full-length cDNA of *GaBLT* or a cDNA fragment of *STI* (encoding the N-terminal 454 amino acids) were transferred to the yeast two-hybrid vectors pGADT7-Rec and pGBKT7 (Clontech) and used to transform the yeast strain AH109 (Clontech).

Co-immunoprecipitation was performed similarly to as described by Staub *et al.* (1996) on *35S::STI*+*35S::GaBLT*-*GFP* transgenic callus. Immunoprecipitation of the GaBLT–green fluorescent protein (GFP) protein was performed using an anti-GFP antibody (Santa Cruz Biotechnology). Protein G–agarose (Sigma) was used to precipitate the immunoprotein complexes. STI detection was performed with an anti-STI antibody. The anti-STI polyclonal antibody was raised against the N-terminal 454 amino acids of STI purified in recombinant yeast *Pichia pastoris*. Fibre protein extraction and western blot analysis were performed as described by Zhao *et al.* (2009). A 10 µg aliquot of proteins was separated in a 12% (w/v) SDS–polyacrylamide gel.

### Bombardment of cultured cotton ovules, cotton transformation, and callus induction

Ovule culture and particle bombardment were conducted as previously described (Hof and Saha, 1997; H.J. Kim *et al.*, 2002; Wang *et al.*, 2010). Ovules of *G. hirsutum* MD51 ne at 2 DPA were bombarded, and then incubated in solid Beasley and Ting medium supplemented with 2.0 µM indole-3-acetic acid (IAA) and 2.0 µM gibberellic acid-3 (GA_3_) for 2 d. Thereafter, the cotton ovules were transferred onto liquid medium containing the same components.

The experimental procedures of *G. arboreum* DPL971 transformation and callus induction were mainly as described by Shang *et al.* (2009). The co-cultivation medium (pH 5.8) was as described in Smith *et al.* (1977). The subculture medium contained naphthaleneacetic acid (2.0mg l^−1^) and benzyladenine (0.5mg l^−1^). Vigorous calli were then selected and transferred onto fresh medium every 4 weeks until the transgenes were detected.

### Cytology

Ovules were removed from the suspension medium and fixed in methanol:glacial acetic acid (3:1). Histochemical localization of β-glucuronidase (GUS) activity was performed according to the protocol reported by Jefferson *et al.* (1987). Small tufts of fibres were removed from the blue-stained GUS-expressing region. Fibre cells were separated using a microspectrophotometer based on the presence or absence of branching. *In situ* nuclear staining was as described by Hof and Saha (1997). The method for fibre nuclear segregation was as described above. Slide preparations and chromosome observation with 4’,6-diamidino-2-phenylindole (DAPI) staining were as previously described (Wang *et al.*, 2007; Gan *et al.*, 2011). The experimental procedures for callus histological observation (in 10 µm thick sections) was as described by Shang *et al.* (2009).

Sequence data in this article are presented with GenBank accession numbers, unless otherwise stated.

## Results

### Identification and cloning of homologous trichome-branching genes in *G. arboreum*

Based on known mechanisms in *Arabidopsis*, an attempt was made to identify homologues of *SPK1*, *KTN1*, *ETR2*, *AN*, *ZWI*, *SCD1*, *KIS*, *POR*, *BLT*, and *STI* in diploid *G. arboreum*. Putative full-length cDNAs of *GaKTN1-1* (KC246031), *GaKTN1-2* (KC246032), *GaETR2-1* (KC246027), *GaETR2-2* (KC246028), *GaETR2-3* (KC246026), *GaKIS* (KC246029), and *GaPOR* (KC246030) were recovered based on *Gossypium* expressed sequence tags (ESTs; from GenBank) and the rapid amplification of cDNA ends (RACE) method. The homologues shared >50% overall sequence identity. All of them had conserved functional domains required for their particular biochemical reactions.

Because no highly homologous sequences of *STI*, *SPK1*, *ZWI*, *AN*, *SCD1*, and *BLT* for *Gossypium* were present in public databases, the *Arabidopsis* mRNA sequences were used to search against raw uncombined *G. raimondii* genome sequences. Primers were designed based on the most frequently identified combined short sequences (~100bp), and gene-containing fragments were amplified from *G. arboreum* genomic DNA or cDNA. Six potential genes were identified [designated *GaSTI* (JQ867271), *GaAN* (JQ711138), *GaBLT* (JQ711139), *GaZWI* (JQ711141), *GaSPK1* (JQ711140), and *GaSCD1* (KC246035)], and their deduced coded proteins all showed >70% identity to corresponding proteins in *Arabidopsis*. To ensure that other gene homologue sequences were not missed owing to the incomplete genome sequence or through base errors, the five sequences were evaluated using low- and high-stringency Southern blots. Only one hybridization band appeared in each lane under high-stringency conditions when using *G. arboreum* sequence probes (Supplementary Fig. S1 available at *JXB* online). These hybridization bands also appeared in the same location under low-stringency conditions when *Arabidopsis*-related sequences were used as a probe. This indicated that single copies of the five *Arabidopsis* homologous genes related to trichome branching had been identified in diploid *G. arboretum* (see the Materials and methods). However, these genes did not result in fibre branching in *G. arboretum*, and hence their expression patterns were investigated.

### *GaBLT* is not expressed in fibres, and cotton *STI* is a pseudogene

The function of a gene is based upon its appropriate expression. The transcriptional levels of all the homologues of the 10 *Arabidopsis* genes were examined with northern blotting. The transcripts of *GaKTN1-1*, *GaKTN*-*2*, *GaETR2-1*, *GaETR2-2*, *GaETR2-3*, *GaKIS*, *GaPOR*, *GaSPK1*, *GaZWI*, *GaAN*, and *GaBLT* were detected in the 3–25 DPA ovule mixture (Supplementary Fig. S2A at *JXB* online). The expression of *GaKIS*, *GaPOR*, *GaSPK1*, *GaZWI*, and *GaAN* occurred at similar levels in fibres. *GaKTN1*, *GaETR2*, and *GaSCD1* mRNA accumulated at significantly higher levels in fibres than in ovules. *GaBLT* transcripts were undetectable in fibres, and this was further confirmed with *in situ* hybridization (Supplementary Fig. S2B). *GaSTI* transcripts were not detected in the ovules or fibres. The presence of *GaSTI* transcripts in other tissues (cotyledon, hypocotyl; root, stem, leaf, at 5- and 15-leaf stages; petal and stamen mixture) was also tested. However, the transcripts were undetectable with northern blotting using seven different probes corresponding to its conceptual reading frame, or by quantitative RT–PCR (results not shown; see the Materials and methods and Supplementary Fig. S3). The above experiments were repeated using *G. hirsutum* RNA samples, and, again, no *STI*-like transcript was detected (data not shown). Thus, *G. hirsutum STI* and *G. arboreum STI* are considered pseudogenes (*ψ*). However, with the exception of *ψGaSTI* and *GaBLT*, the other identified genes were expressed in fibres, suggesting that fibre cells may have the potential for branching.

### Nuclear DNA content of the fuzz fibre is constant, and a limited and reversible change occurs in lint

To test the possibility that the absence of *GaBLT* expression affects fibre endoreduplication levels, the nuclear DNA content of *G. arboreum* DPL971 fibres at 20 and 25 DPA was examined using the 2C criterion for a root tip nucleus. At 20 and 25 DPA, lint nuclear DNA averaged 3.3±0.28 pg (*n=*582) and 3.4±0.3 pg (*n=*547), whereas fuzz DNA content averaged 3.4±0.3 pg (*n=*510) and 3.5±0.25 pg (*n=*563), respectively. These results closely correspond to root tip measurements [3.4±0.25 pg, *n*=479; *P >* 0.05, Kruskal–Wallis one-way analysis of variance (ANOVA) on ranks and an all pairwise multiple comparison Dunn’s test].

The constant nuclear DNA content in mature fibre cells that had ceased elongation (~20 DPA) may prolong the early developmental stage that is amenable to division. In this case, the timing of endoreduplication may be altered in the early stages of fibre development, followed by degradation or other event(s) leading to the observed 2C level. Unfortunately, accurate measurement of DNA content from early developmental stages of fuzz fibres is difficult because the fuzz fibres are concealed amongst lint fibres. Consequently, as a proxy for detecting endoreduplication levels at the early fuzz developmental stages, the differences in fibre DNA content were compared between DPL971 (containing both lint and fuzz) and its fuzzless lint-normal mutant DPL972. Histological sections revealed that the fuzz of DPL971 was initiated at 3–4 DPA in our cultivation conditions (Wang *et al.*, 2013). Nuclei from the fibres of both strains exhibited elevated DNA content (~3.4 pg, 2C to ~4.1 pg, 2.4C) of the same order of magnitude from 2 to 4 DPA (Supplementary Fig. S5A at *JXB* online). The statistical mean of the nuclear DNA content was slightly reduced from ~4.1 pg, 2.4C (4 DPA) to 3.8 pg, 2.2C (7 DPA) in DPL971. In contrast, 5 or 7 DPA DPL972 nuclear DNA content was equal to that at 4 DPA. These results demonstrate that the fuzz nuclei had a lower nuclear DNA content, which lowered the statistical mean nuclear DNA content of DPL971 samples that inevitably also contained fuzz nuclei. Furthermore, in the wild-type DPL971, the percentage of 5 and 7 DPA nuclei with the highest relative fluorescence units (RFU, 2.0) was lower than at 4 DPA, and the percentage of nuclei with 1.6 RFU (2C) was increased (Supplementary Fig. S5A). Thus, the increased proportion of 2C nuclei was attributed to fuzz because this profile did not appear in the nuclear determination of fuzzless DPL972 (Supplementary Fig. S5A). The above results indicated that fuzz DNA was constant (2C) at the fuzz fate determination and initiation stages, and elevated in lint.

The timing around lint initiation was also accessed. Swelling of the trichoblast cells that initiated lint growth occurred at –2 to –1 DPA in DPL971 (Supplementary Fig. S4 at *JXB* online). A laser microdissection system was used to collect adjacent epidermal cells from the DPL971 ovule at –4 to –5, –3, and –2 DPA) and from the lint and surrounding cells (at –1, 0, and 1 DPA). The DNA levels in epidermal cells did not differ from those of root tip cells before the occurrence of the swelling of the trichoblast. No detectable differences were found between the lint cells and the surrounding unexpanded cells (Supplementary Fig. S5B). This indicates that DPL971 lint fibre DNA content was constant before 1 DPA.

To summarize, the DNA content of fuzz cells was constant at the 2C level when endoreduplication did not occur. Upon initiation, an ephemeral DNA partial reduplication (note, it is unsure if it is an endoreduplication; see Discussion) was observed in lint, followed by a return to the 2C level.

### Overexpression of *GaBLT* increases cotton cell endoreduplication levels, but co-expression with interactive *Arabidopsis STI* made no additional contribution to the DNA cycle

**Fig. 1.**
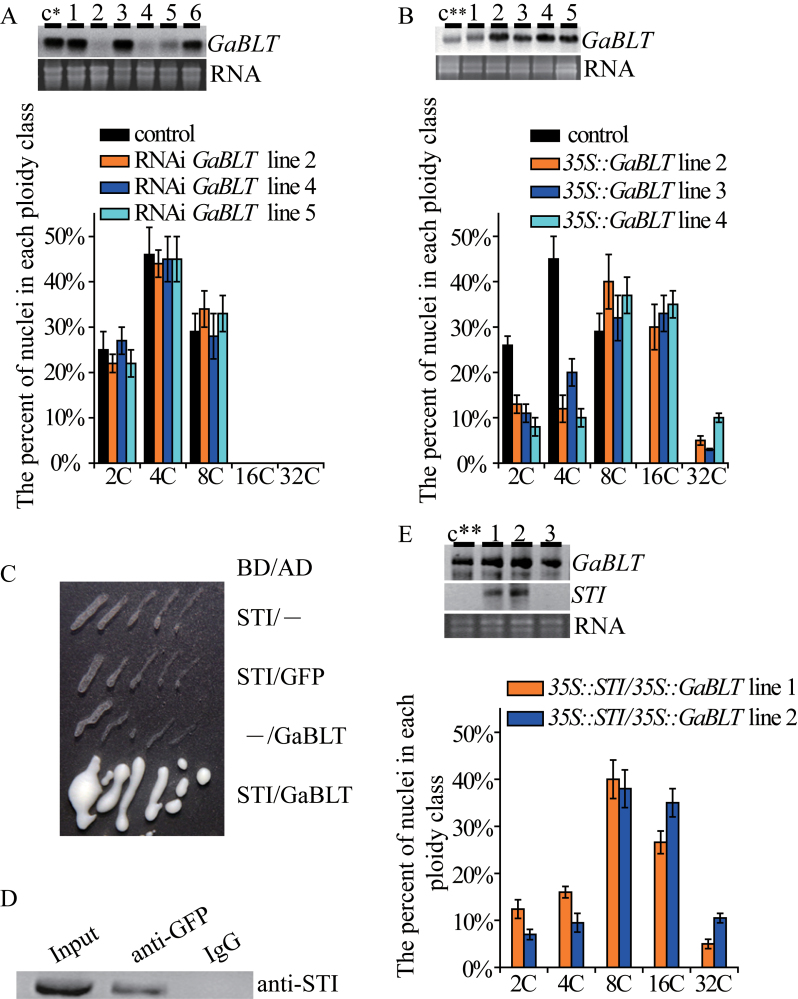
The effects of *GaBLT* epistasis and overexpression on endoreduplication levels. (A) Northern blot with an antisense *GaBLT* probe showing *GaBLT* mRNA in independent *GaBLT*-RNAi cell lines (top panel). Flow cytometric DNA analysis of the effectual *GaBLT*-RNAi cell lines (lower panel). (B) Northern blot showing *GaBLT* mRNA in independent *35S::GaBLT* transgenic cell lines (top panel). Flow cytometric DNA analysis of the effectual *35S::GaBLT* cell lines (lower panel). (C) Interaction between STI and GaBLT in a −His +3-amino-1,2,4-triazole (30mM) plate. BD, GAL4-binding domain; AD, GAL4 activation domain. Note, STI and GFP do not interact. (D) Co-immunoprecipitation of GaBLT–GFP and STI. Transgenic callus extracts were immunoprecipitated with an anti-GFP antibody and detected with western blotting using an antibody raised against STI. IgG, pre-immune serum. (E) Northern blotting with an antisense *GaBLT* probe showing *GaBLT* mRNA in independent *35S::GaBLT* and *35S::STI* co-transformed cell lines (top panels). Flow cytometric DNA content analysis of *GaBLT* and *STI* co-expressing cell lines (lower panel). The control c* or c** lines were transformed with the pART27 or pBI121 empty vectors, respectively. DNA content of the non-transgenic cell lines was the same as the lines transformed with pART27 or pBI121. Error bars in (A), (B), and (E) indicate the SD for three independent experiments. The numbers in the top images of (A), (B), and (E) indicate cell lines.

*GaBLT* was expressed in cotton callus cells (Fig. 1A, B). To test if *GaBLT* was essential for endoreduplication in cotton, RNAi (RNA interference) lines were generated in which the interfering RNA was targeted specifically to the 3’-coding region of *GaBLT.* Six independent transgenic cell lines were analysed with northern blotting for *GaBLT* transcripts. Three of these RNAi cell lines had significantly low levels of *GaBLT*, but no effect on nuclear DNA content (Fig. 1A), suggesting that *GaBLT* function was not required for endoreduplication. However, all transgenic cell lines overexpressing *GaBLT* driven by the *35S* promoter showed a significant increase in DNA content relative to untransformed cell lines or pBI121 empty vector-transformed cell lines (Fig. 1B). Therefore, *GaBLT* was sufficient for endoreduplication in cotton callus cells.

In *Arabidopsis*, *BLT* interacts both genetically and physically with *STI* (Kasili *et al.*, 2011). The hypothesis that GaBLT and STI proteins interact was tested using two methods. Yeast two-hybrid experiments demonstrated a clear interaction between full-length GaBLT and the N-terminal 472 amino acids of *Arabidopsis* STI (Fig. 1C). The interaction between STI and GaBLT *in vivo* was confirmed with an immunoprecipitation experiment in which the full-length GaBLT coding region was fused to *GFP* and co-expressed with *STI* in callus cells (Fig. 1D). The two protein products may act as part of a complex and induce endoreduplication. However, the nuclear DNA content of the cell lines co-expressing *STI* and *GaBLT* did not differ significantly from that of the *35S::GaBLT* transgenic cell lines (Fig. 1B, E; *P* > 0.05, Kruskal–Wallis one-way ANOVA on ranks and Dunn’s test). The above results indicated that ectopic STI expression may have a specific function in conjunction with *GaBLT* in cotton, but that this role is not endoreduplication. Interestingly, the homologue of *GaBLT* in *G. hirsutum* was expressed in the cultured fibres (subsequent result in Supplementary Fig. S7 at *JXB* online), indicating that *GaBLT* alone was not enough to promote fibre branching and that an interactive *STI* homologue or an analogous factor may be necessary.

### Overexpression of *GaSIM* promotes cotton cell transition from mitosis to endoreduplication

Mitosis occurs in differentiated fibre cells of cultivar MD51 ne ovules grown in *in vitro* culture (at 2 DPA, at a high temperature of 34 °C), producing multicellular, unbranched fibres harbouring individual nuclei in each cell (Hof and Saha, 1997, 1998). Trichomes of the *Arabidopsis sim* mutant are also multicellular and have normal morphology. SIM inhibits transition from the G_2_ phase to mitosis and contributes to endoreduplication (Churchman *et al.*, 2006).

**Fig. 2.**
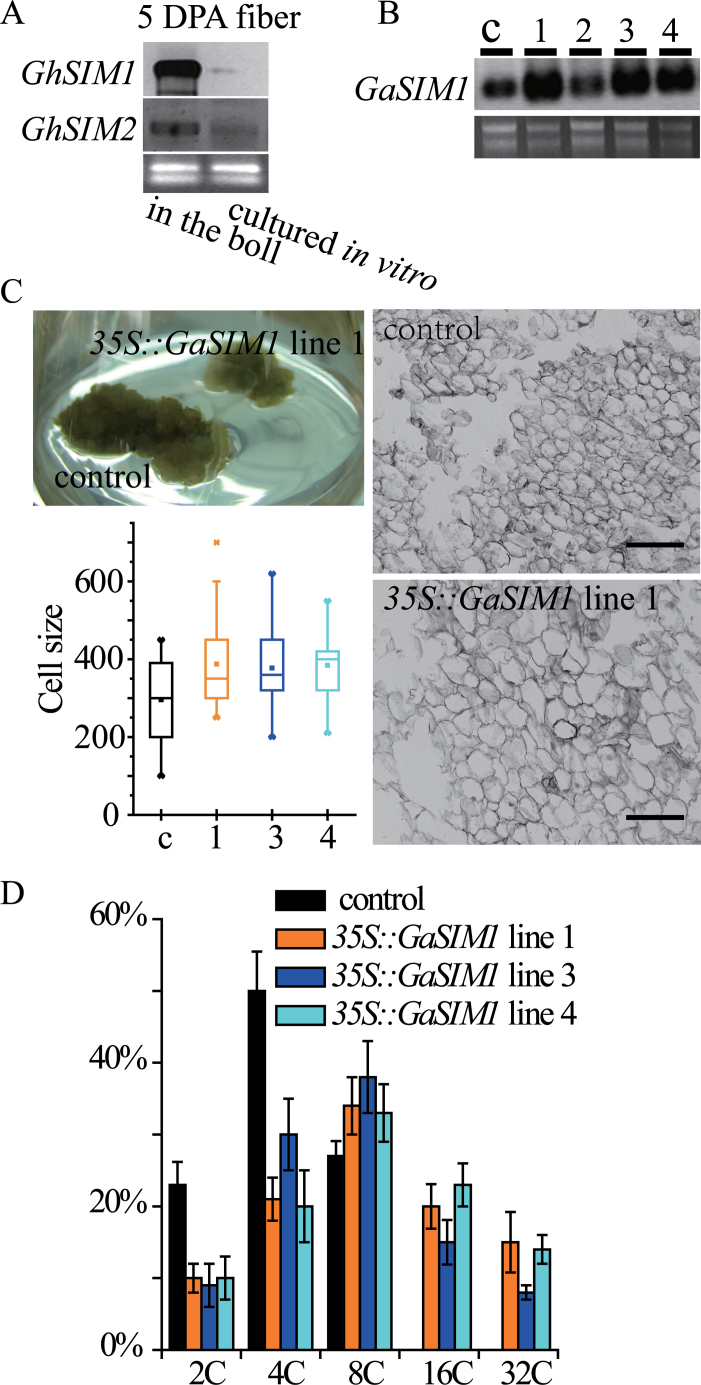
Cotton *SIM* plays a role in repressing cytokinesis. (A) Northern blotting with antisense *GaSIM1* and antisense *GaSIM2* probes showing *GhSIM1* and *GhSIM2* mRNA levels in 5 DPA fibres of the *G. hirsutum* cultivar MD51 ne. The 5 DPA fibres came from *in vivo* (in the boll) or *in vitro* cultures (2 DPA ovules were cultured for 3 d). (B) Northern blot showing *GaSIM1* mRNA levels in four independent *35S::GaSIM1* cell lines and the control (c). (C) Upper left panel: the phenotype of a cell line overexpressing *GaSIM1* and a pBI121 empty vector (control) transformed cell line after two successive culture transfers. Lower left panel: cell size (µm^2^, area inside of the perimeter) of the callus. Calli were dispersed with water, and the cells were quantified with ImageJ software. The *35S::GaSIM1* lines differed significantly from the control (transformed with pBI121 empty vector) (*P* < 0.05, Kruskal–Wallis one-way ANOVA on ranks and Dunn’s test). Data are presented as box plots in which the box encompasses the 25th to the 75th percentile of the data, the line within the box is the median (50th percentile), and the error bars represent the 5th (lower bar) and the 95th (upper bar) percentiles. Right panels: representative micrographs of the transgenic and control cell lines. Bar=40 µm. (D) Nuclear DNA content of *35S::GaSIM1* cell lines and control (transformed with pBI121) as determined with flow cytometry. The percentage of nuclei in each ploidy class is indicated. Error bars indicate the SD for three independent experiments.

To assess the correlation between the multicellular phenotype and endoreduplication, two *SIM* homologous sequences in *G. arboreum* [*GaSIM1* (KC246033) and *GaSIM2* (KC246034)] were cloned based on the uncombined genome sequence of *G. raimondii*. Predicted GaSIM1 and GaSIM2 had all conserved motifs (Supplementary Fig. S6 at *JXB* online), suggesting a similar function to that of other members of the SIM family (Churchman *et al.*, 2006). Next, an attempt was made to investigate the expression of *GaSIM* genes in cultured fibres, but producing elongated fibres from *G. arboreum* ovules was difficult. Hence, northern blotting was performed with probes corresponding to *GaSIM1* and *GaSIM2* using *G. hirsutum* fibre RNA samples. Transcription of both *G. hirsutum SIM* genes was significantly reduced in cultured fibres (Fig. 2A) which harboured multicellular fibres (see Supplementary Fig. S8; Hof and Saha, 1997, 1998).

To investigate further the biological function of *GaSIM* genes, transgenic calli overexpressing *GaSIM1* from the *35S* promoter were created. The three positive transgenic *35S*::*GaSIM1* lines (Fig. 2B, lanes 1, 3, and 4) displayed a similar phenotype, showing a reduction in overall callus size and increased cell size compared with the control (transformed with empty pBI121) (Fig. 2C). These results suggest that cell division was reduced in lines overexpressing *GaSIM1*. Flow cytometric analyses confirmed that the *35S::GaSIM1* lines had extra rounds of endoreduplication, with increased levels of 16C and 32C cells detected (Fig. 2D). Based on the above results, it is inferred that GaSIM1 functions in repressing cotton cell cytokinesis, thus causing the transition from mitosis to endoreduplication.

### Co-expression of *GaBLT*, *GaSIM*, and *STI* in *in vitro* cultured fibres causes branching

**Fig. 3.**
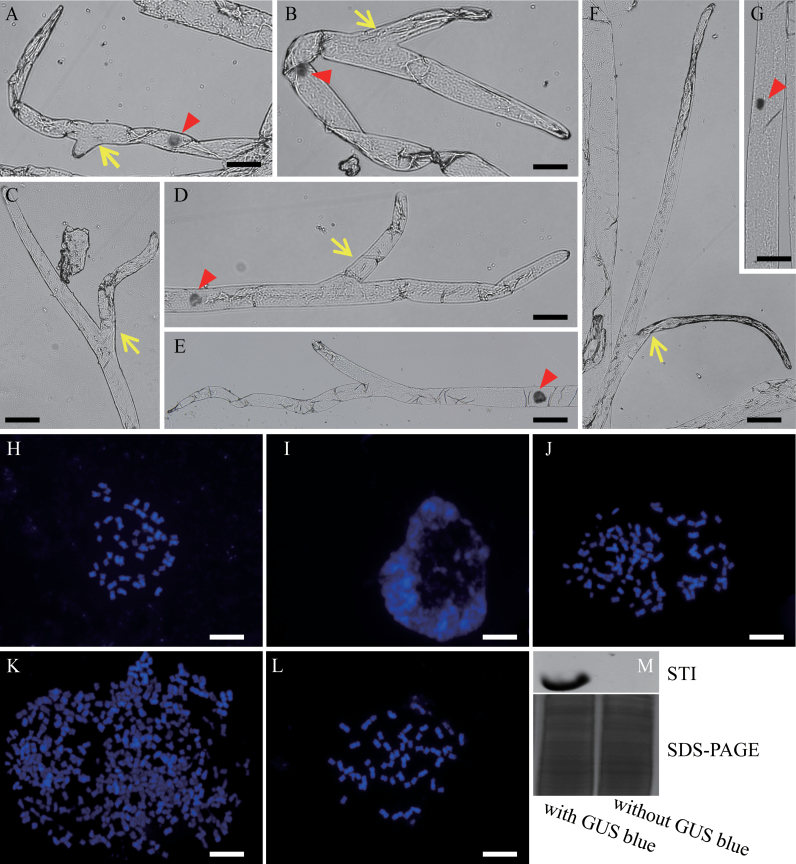
Co-expression of *GaBLT*, *GaSIM*, and *STI* can induce the fibre-branching phenotype. Ovules were collected at 2 DPA and bombarded with a construct to express *GaBLT*, *GaSIM1*, *Arabidopsis STI*, and *GUS* in fibres, followed by *in vitro* culture for 5 d. Branched fibres showing transgene expression as indicated by GUS staining were rinsed with water to reduce GUS staining, followed by nuclear staining with Schiff’s reagent (A–F) or separation of the nuclei and staining of the chromosomes with DAPI (J and K). (A– F) Branched fibres. (G) A bombarded fibre without GUS staining (branchless) as a negative control to see the increase in size of th enucleus in branching fibres. The fibre was also stained with Schiff’s reagent. (H) Metaphase chromosomes of a bombarded fibre without GUS staining. (I) Chromosome phenotype of a 7 DPA untreated fibre constantly growing *in vivo* (in the boll). (J) Chromosome phenotype of a bombarded but branchless fibre cell with GUS staining. (K) Chromosome phenotype of a bombarded and branched fibre cell with GUS staining. (L) Metaphase chromosomes of a *G. hirsutum* root tip cell. (M) Western blot showing *Arabidopsis* STI protein levels in fibres with or without GUS staining. Yellow arrows show branching sites; red arrowheads show cell nuclei. Bars=50 µM (A–G) or 10 µM (H–M).

Cotton fibres are completely branchless in wild-type plants. Extensive scanning electron microscope observations of a large number of samples did not reveal any branching in fibres cultured *in vitro*. The transgenic fibres were then examined using the cotton ovule culture/biolistic transformation system, which is a quick and effective expression assay for detecting gene function and promoter specificity in fibres (H.J. Kim *et al.*, 2002; Li *et al.*, 2007; Wang *et al.*, 2010). Northern blot analyses showed that the homologues of *GaKTN1-1*, *GaKTN1-2*, *GaETR2-1*, *GaETR2-2*, *GaETR2-3*, *GaKIS*, *GaPOR*, *GaSPK1*, *GaAN*, *GaZWI*, and *GaSCD1* were expressed in cultured *G. hirsutum* fibres (data not shown). An attempt was made to take advantage of (i) fibre nuclear DNA amplification processes circumstantially *in vitro*; (ii) the specific Ga*SIM1* promoter of the mitosis to endoreduplication transition; and (iii) *Arabidopsis STI* functioning as a substitute for the cotton pseudogene. A *GaBLT*, *GaSIM*, and *Arabidopsis STI* expression construct was created, with the three genes being driven by the cotton fibre-specific promoter *SCFP*, which is activated at 0–25 DPA (Hou *et al.*, 2008; Zhang *et al.*, 2011). The construct also contained a *SCFP::GUS* reporter gene, which allowed visual identification of transformed fibre cells. *GUS* was expressed in some fibre cells (a tuft) 5 d after bombardment with the construct at 2 DPA, indicating successful transgene expression in these fibres (Supplementary Fig. S7A, B at *JXB* online). Interestingly, 12.3% (*n*=560) of the GUS-stained tufts had two-branched fibres that were shorter and thinner than non-transformed fibres (Fig. 3A–F; Table 1).

**Table 1.** Impact on cotton fibre branching following bombardment of cultured ovules with overexpression constructs containing the GUS reporter gene

Ovule age when placed into culture	Bombarded genes overexpressed in fibres	No. of branched fibres	Total no. of GUS-stained fibre tufts
2 DPA	*STI+GUS*	0	93
2 DPA	*GaSIM1+GUS*	0	89
2 DPA	*GaBLT+GUS*	0	78
2 DPA	*GaBLT*+*STI+GUS*	0	91
2 DPA	*GaBLT*+*GaSIM1+GUS*	0	101
2 DPA	*GaSIM1*+*STI+GUS*	3	483
0 DPA	*GaSIM1*+ *STI+GUS*	0	494
2 DPA	*GaBLT*+*GaSIM1+STI+GUS*	69	560

The branched and branchless fibres were captured using the laser microdissection system. Nuclear staining revealed that the GUS-stained fibres were all monocytes (Fig. 3A, B, D, E; *n*=252), with a larger nucleus (compared with a normal nucleus in Fig. 3G), in contrast to multicellular non-transgenic fibres (Supplementary Fig. S8 at *JXB* online), demonstrating the positive function of GaSIM. The impact of the transgene on branched and branchless fibres was then assessed by comparing the quantity of DNA with the chromosome number. The number of chromosome in the ovules supplier *G. hirsutum* is 52 (Wang *et al.*, 2007). During metaphase, fibres lacking GUS staining had 52 chromosomes (Fig. 3H). Compared with the 5 DPA fibre cells grown *in vivo*, which had no separated chromosomes during interphase (Fig. 3I), >52 chromosomes were observed in the GUS-positive fibres growing *in vitro* (Fig. 3J, K), and the chromosome phenotype was similar to that of root tip cells (Fig. 3L). This indicated the occurrence of endoreduplication in the GUS-positive blue-stained fibres. Branchless fibres positively stained with GUS had no more than 104 chromosomes (4C, *n*=20), whereas branched fibres had up to 516 chromosomes (Fig. 3K; 16C, *n*=14). The above data showed that the DNA content of the transgenic fibres was elevated, attaining up to 16C when branching occurred. Additionally, ectopic expression of *Arabidopsis* STI protein in the GUS-stained fibre tufts was confirmed with western blotting (Fig. 3M).

To determine if all three genes (*GaBLT*, *GaSIM1*, and *STI*) functioned in fibre branching, different combinations of the genes and the GUS reporter were bombarded. Individually, none of the three genes promoted fibre branching (Table 1). Combinations of *GaBLT*+*GaSIM1* or *GaBLT*+*STI* did not promote fibre branching (Table 1), successively indicating the necessity of *STI* and *GaSIM1*. However, a rare occurrence (0.6%) of branched fibres was observed in *GaSIM1+STI* construct-bombarded GUS-stained tufts (Table 1). This was a consequence of the autonomous expression of *G. hirsutum BLT* (*GhBLT*) in cultured fibres (Supplementary Fig. S9 at *JXB* online). All of the above data were obtained from fibres cultured beginning at 2 DPA. However, no *GhBLT*-expressing fibres were produced on 0 DPA ovules, which did not undergo fibre cell mitosis following cultivation for 5 d (Beasley and Ting medium with IAA and GA_3_) (Supplementary Fig. S9). When the 0 DPA ovules were bombarded with the *GaBLT*+*STI* construct, no branched fibres were observed (Table 1), indicating the necessity of exogenous *GaBLT*. Thus, the presence of all three genes was essential for branching in cultured fibres.

## Discussion

### Elevated endoreduplication levels are not sufficient for initiating cotton fibre branching

The nucleolus, which is a round or oval large body in the nucleus, consists of DNA (rDNA), RNA, and protein (Thiry and Lafontaine, 2005). The mean nucleolar volume increased rapidly, reached a maximum before 10 DPA, and was followed by a decline, according to varieties (De Langhe *et al.*, 1978; Peeters *et al.*, 1988). Whether the main contributors to enlargement are RNA, DNA, or protein is unclear. Thus, it cannot be confirmed if the lint DNA content elevation (Supplementary Fig. S5 at *JXB* online) was caused by endoreduplication, the nucleolar rDNA increasing [such as accumulation of extrachromosomal rDNA circles (Sinclair and Guarente, 1997)], or both. In addition, micronuclei were found in the fibres at or a little before 4 DPA, and were absent before 2 DPA; in the same variety and cultivation, until 4 DPA (at 2–3 DPA; the authors’ unpublished data), the lint DNA is elevated. So, the previous deduction that DNA content increasing in early developing fibres was due to an enlarged micronucleus (Taliercio *et al.*, 2005) needs to be reconsidered.

In most genotypes, the degree of endoreduplication is highly correlated with that of *Arabidopsis* trichome branching (Hülskamp *et al.*, 1994; Kasili *et al.*, 2011). The *Arabidopsis* trichome undergoes progressive endoreduplication cycles during the early stages of cell morphogenesis, leading to increased nuclear DNA content. Mutations that affect trichome nuclear DNA content also alter trichome branching, with tetraploid lines producing overbranched trichomes compared with diploid lines (Perazza *et al.*, 1999). Cotton fuzz cells were maintained at the G_0_ phase with a constant 2C nuclear DNA content (Supplementary Fig. S5A at *JXB* online), which may be correlated with their unbranched phenotype. Interestingly, no branching occurred while the DNA content of lint fibres increased from 2C to the maximum 2.4C over a 3 d period (2–5 DPA; Supplementary Fig. S5A), possibly because the endoreduplication (if it happened) levels did not attain a required threshold. Prior to the initiation of branching, the trichome nucleus undergoes three rounds of endoreplication (Hülskamp *et al.*, 1994).

BLT has been conserved throughout angiosperm evolution, and a known link exists between the coordination of cell shape and nuclear DNA content in the *Arabidopsis* trichome. Both dicots and monocots have BLT homologues, which is indicative of conservation of their function throughout angiosperm evolution (Kasili *et al.*, 2011). Gene performance and function are dependent on temporal and spatial expression patterns. *GaBLT* is unique in the genome (Supplementary Fig. S1 at *JXB* online); it is not widely expressed in the ovule and is absent in fibres. The overexpression of *GaBLT* in cotton callus cells significantly elevated DNA content (Fig. 1B), suggesting that it is potentially involved in endoreduplication. Differentiated fibres on cultured ovules expressing cotton *BLT* were branchless (Supplementary Fig. S9; Hof and Saha, 1997, 1998), even in the *35S::GaBLT* fibres (Table 1), indicating that *GaBLT* alone was not sufficient to induce cultured fibre branching. Moreover, *GaSIM1* repressed cotton cell cytokinesis (Fig. 2), and co-expression with *GaBLT* in cultured fibres significantly elevated the nuclear DNA content to 16C (~500 chromosomes; data not shown), but branched fibres were not induced (Table 1). Hence, a high endoreduplication level was not sufficient for initiation of cotton fibre branching.

### Cotton fibres have branching potential

*Arabidopsis* stem trichomes are predominantly unbranched (Marks and Feldmann, 1989), with only 10–20% having two branches (Perazza *et al.*, 1999). In contrast, nearly all of the stem trichomes on the *triptychon* mutant display two or three branches (Perazza *et al.*, 1999). Additionally, stem trichomes subjected to *STI* overexpression exhibit two branching points (Ilgenfritz *et al.*, 2003). These reports suggest that branching is controllable, as long as gene expression occurs at appropriate levels. Many *Arabidopsis* homologous branching genes were expressed in fibres (Supplementary Fig. S2A at *JXB* online), except *GaBLT* and *ψGaSTI*. The *Arabidopsis* trichome branching gene *STI* expressed with the endoreduplication inducers *GaSIM* and *GaBLT* in fibres induced branching (Fig. 3A–F). This demonstrated that trichome patterning genes could be used to produce homologous economically useful cotton fibres. The branched fibres may improve bulkiness, modifying batting, ease of hardening, and poor resilience. The branched-fibre changed the fibre physical structure which could improve bulkiness by increasing the gap between each fibre Thus, the branched fibres may be useful for non-woven applications, such as packing material used in quilts and cotton-padded coats.

*Arabidopsis* trichome branching involves a temporally defined sequence of events: endoreduplication, initiation of primary branching, continued endoreduplication, and secondary branching; primary and secondary branching are genetically distinct (Hülskamp *et al.*, 1994; Folkers *et al.*, 1997). The angle between the primary branches and the main stem is ~109 °, whereas it is ~85 ° between secondary branches, and the nucleus is typically positioned at the first branch point (Folkers *et al.*, 1997). Hence, the cotton fibre branching architecture was more similar to the *Arabidopsis* trichome secondary branches based on two criteria: (i) the angle between the two branches was <90 °; and (ii) the nucleus was located below the branching point.

The genomic sequence of *ψGaSTI* showed an intact STI-like reading frame and codon bias, but additional and longer introns than *STI* (Supplementary Fig. S3 at *JXB* online). Whether *ψGaSTI* is a relic or if it is evolving to become functional is not known, but potential genes with the ability to regulate fibre branching in cotton may exist.

### Cotton fibres have a unique identity compared with *Arabidopsis* trichomes and root hairs

Cotton fibres may be unique in that they are not a consequence of endoreduplication. In *Arabidopsis*, undifferentiated cells destined to become pro-trichome cells exit the mitotic cycle and enter into endoreduplication cycles, and the pro-trichome cells can form branches and expand during endoreduplication (Schnittger and Hülskamp, 2002; Ishida *et al.*, 2008). Lint and fuzz showed no detectable endoreduplication before swelling (Supplementary Fig. S5 at *JXB* online), meaning that the endocycle was not an essential event for fibre initiation.

IAA (natural auxin) accumulates in cotton fibre initials. Genetic engineering to increase IAA levels in the ovule epidermis at the fibre initiation stage leads to a substantially increased number of lint fibres (Zhang *et al.*, 2011). In addition, IAA is required for fibre production during *in vitro* culture of unfertilized ovules (Beasley, 1973; Beasley and Ting, 1973). These findings suggest that IAA is beneficial for fibre development. *Arabidopsis* root hairs, which are unbranched single cells, share a similar cell fate determination pathway (substrate depletion and lateral inhibition) with trichomes (Hülskamp, 2004; Ishida *et al.*, 2008). IAA can induce root hair but not trichome production, and is not involved in the regulation of substrate depletion and the lateral inhibition pathway (Masucci and Schiefelbein, 1996; Rahman *et al.*, 2002). This pathway acts upstream of, or independently from, the auxin pathway (Masucci and Schiefelbein, 1994, 1996; Ishida *et al.*, 2008). High IAA levels have a negative effect on endoreduplication, and mutants in IAA signalling, biosynthesis, or transportation show an increased final DNA ploidy level (Ishida *et al.*, 2010). Thus, two antagonistic pathways exist, and both are able to promote *Arabidopsis* root hair development. If substrate depletion and the lateral inhibition pathway play a role in fibre development, as mentioned previously, then we may reasonably conclude that fibres are similar to root hairs. However, hair cells require a lower active IAA supply than non-hair cells (Jones *et al.*, 2008). Therefore, based on the present results, cotton fibres are not identical to *Arabidopsis* root hairs or trichomes, although they do share some homology. Fibres must have a regulatory mechanism that is not dependent on endoreduplication.

## Supplementary data

Supplementary data are available at *JXB* online.

Supplementary methods.

Figure S1. Southern blots with high stringency and low stringency.

Figure S2. Characteristics of the homologous genes controlling *Arabidopsis* trichome branching in *G. arboreum.*

Figure S3. Genomic sequence of *ψGaSTI*.

Figure S4. Histological observation of DPL971 seeds at different developmental stages.

Figure S5. Fibre nuclear DNA content at different developmental stages.

Figure S6. Alignment and evolutionary tree of GaSIM-related plant proteins.

Figure S7. Co-expression of *GaBLT*, *GaSIM*, and *STI* can induce fibre-branching phenotype.

Figure S8. Fibres with multicelled and unicelled phenotype.

Figure S9. Northern blots with antisense *GaBLT* probe showing *GhBLT* mRNA levels in cultured fibres.

Supplementary Data
