# Supplementary material for: Induction of cotton ovule culture fibre branching by co-expression of cotton BTL, cotton SIM, and Arabidopsis STI genes
Source: J Exp Bot. 2013 Aug 21;64(14):4157–68. doi: 10.1093/jxb/ert222 (PMC3808306; doi:10.1093/jxb/ert222)
Supplement: Supplementary Data [file supp_64_14_4157__index.html]

Induction of cotton ovule culture fibre branching by co-expression of cotton BTL, cotton SIM, and Arabidopsis STI genes — Induction of cotton ovule culture fibre branching by co-expression of cotton BTL, cotton SIM, and Arabidopsis STI genes — Supplementary Data 

# Induction of cotton ovule culture fibre branching by co-expression of cotton *BTL*, cotton *SIM*, and *Arabidopsis STI* genes

## 

Data files

**Files in this Data Supplement:**

- Supplementary Data - Supplementary Data
