# Supplementary material for: Induction of cotton ovule culture fibre branching by co-expression of cotton BTL, cotton SIM, and Arabidopsis STI genes
Source: J Exp Bot. 2013 Aug 21;64(14):4157–68. doi: 10.1093/jxb/ert222 (PMC3808306; doi:10.1093/jxb/ert222)
Supplement: Supplementary Data [file supp_ert222_jexbot099911_file001.pdf]

Supplementary data

Induction of cotton ovule culture fiber branching by a combination of three genes of *BRANCHLESS TRICHOMES, SIAMESE1,* and *STICHEL*

Gaskin Wang, Hong-Jie Feng, Jun-Ling Sun, Xiong-Ming Du

State Key Laboratory of Cotton Biology, Institute of Cotton Research of Chinese Academy of Agricultural Sciences, Anyang 455000, Henan Province, China

Supplementary Methods

Plasmid constructs

Both of the two *CaMV35S* (*35S*) promoters in pCAMBIA1301(CAMBIA) were replaced by the *SCFP* promoter (Hou et al., 2008). The *GUS* in pCAMBIA1301 was replaced by *GaSIM1* cDNA at the *Nco*I–*Nhe*I site; the cDNA of *Arabidopsis STI* cDNA was cloned into the *Xho*I site (to replace the hygromycin resistance gene), forming *SCFP::STI:35S poly A*/*SCFP::GhBLT:Nos poly A*. The *CaMV35S* promoter of pBI121 was replaced by *SCFP* promoter at the *Hind*III–*Xba*I site, forming *SCFP::GUS* construct, then the GUS coding region was replaced by *GaBLT* cDNA. PCR was performed to amplify the fragment containing *SCFP* promoter, *GaBLT* cDNA, and *Nos poly A* terminator. Next, the fragment was inserted into *SCFP::STI:35S poly A*/*SCFP::GaSIM1:Nos poly A* at the *Kpn*I site, forming *SCFP::STI:35S poly A*/*SCFP::GhBLT:Nos poly A/SCFP::GaSIM1:Nos poly A*. The *SCFP::GUS: Nos poly A* region of the *SCFP::GUS* construct was obtained using PCR, and then the PCR fragment was inserted into the above construct at the *Sma*I site, forming *SCFP::STI:35S poly A*/*SCFP::GhBLT:Nos poly A/SCFP::GUS:Nos poly A/SCFP::GaSIM:Nos poly A.*

The intron-containing hairpin (ihp) construct with the pHANNIBAL/pART27 system was used to create RNA interference (RNAi) (Wesley et al., 2001). To produce transgenic cells expressing a *GaBLT*-RNAi construct, a 500-nucleotide *GaBLT* C-terminal coding region was amplified. The PCR product was cloned in both sense and antisense directions into pHANNIBAL. The construct was then subcloned into pART27, and introduced into *Agrobacterium* strain LBA4404.

The entry clone of full-length *GaBLT* was fused with the N-terminal of GFP in the pCAMBIA1302 (CAMBIA) vector. Full-length *Arabidopsis STI* replaced the GUS coding region of pBI121, then the PCR fragment (with supernumerary *Eco*RI recognition site at two ends) of *35S::STI:NOS-ter* was subcloned into the intermediate pCAMBIA1302 at the *Eco*RI by the use of In Fusion technology (Clontech) to create *35S::STI-35S::GaBLT-GFP.*

Hou L, Liu H, Li JB, Yang X, Xiao YH, Luo M, Song SQ, Yang GW, Pei Y. 2008. SCFP, a novel fiber-specific promoter in cotton. *Chinese Science Bulletin* **53**, 2639-2645.

Wesley SV, Helliwell CA, Smith NA, Wang MB, Rouse DT, Liu Q, Gooding

PS, Singh SP, Abbott D, Stoutjesdijk PA. 2001. Construct design for efficient, effective and high‐throughput gene silencing in plants. *The Plant Journal* **27**, 581-590.

Supplementary figures

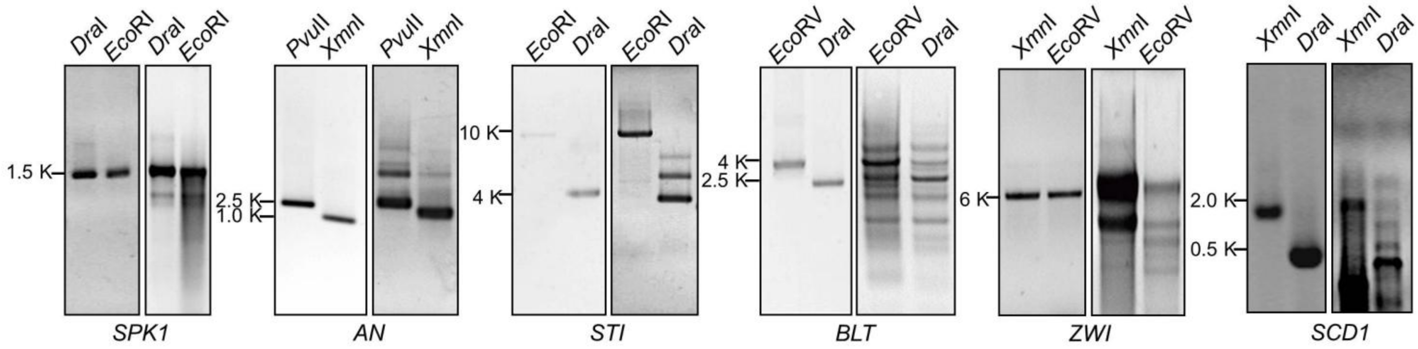

Fig. S1. Southern blots with high stringency (on the left side of each pair box, the probes are strongly complementary to *G. arboretum*-related genes) and low stringency (on the right side of each pair box, the probes are strongly complementary to *Arabidopsis*-related genes), respectively. Relative sizes of the hybridizing bands are indicated in kb (K).

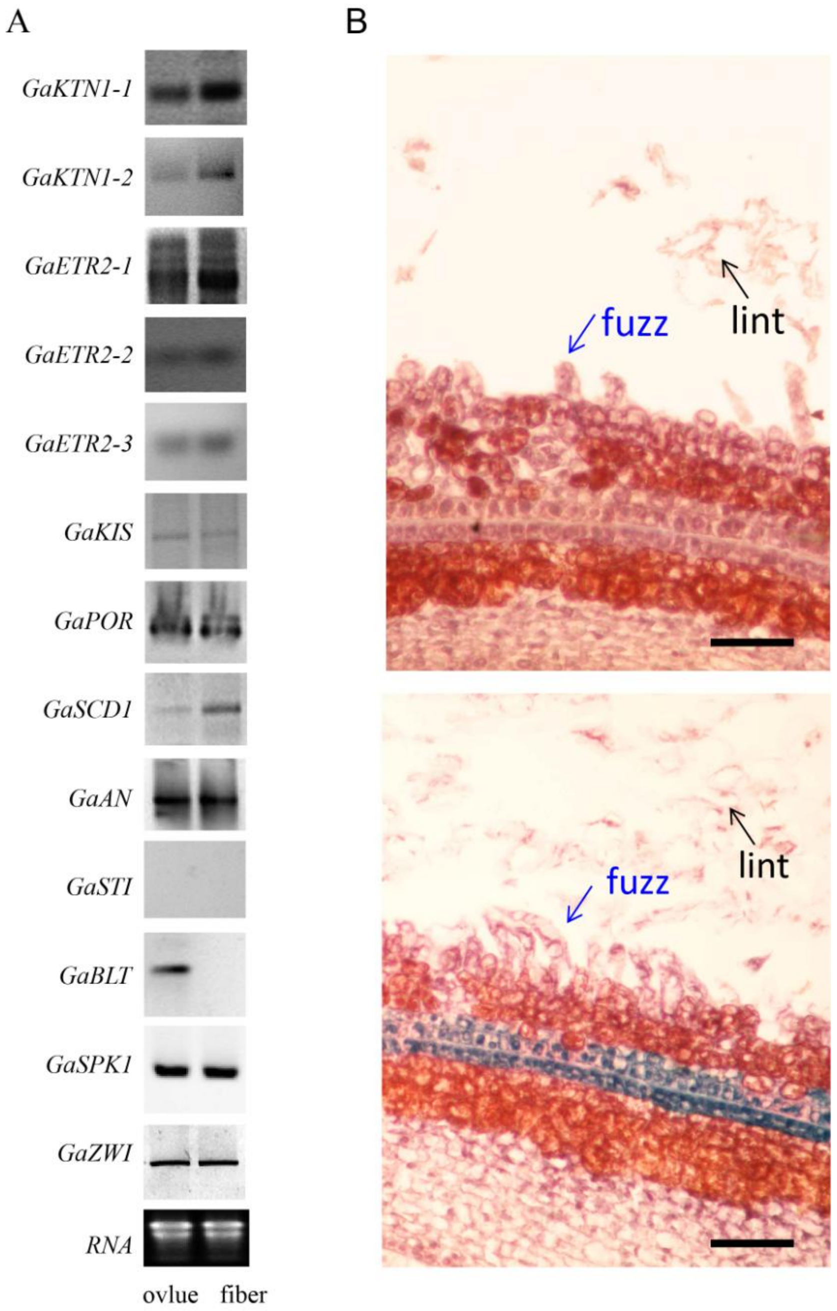

Fig. S2. Characteristics of the homologous genes controlling *Arabidopsis* trichome branching in *G. arboreum*.

(A) Northern blots showing genes expression in fibers and ovules. RNA used in the analysis was derived from a balanced mix of total-RNA samples from 3 to 25 DPA fibers or ovules.

(B) Longitude-sections of 5 DPA cotton seed hybridized with sense (up) and antisense (down) RNA probes for *GaBLT*. Blue-dark indicates hybridization signal. Bars = 20 μm

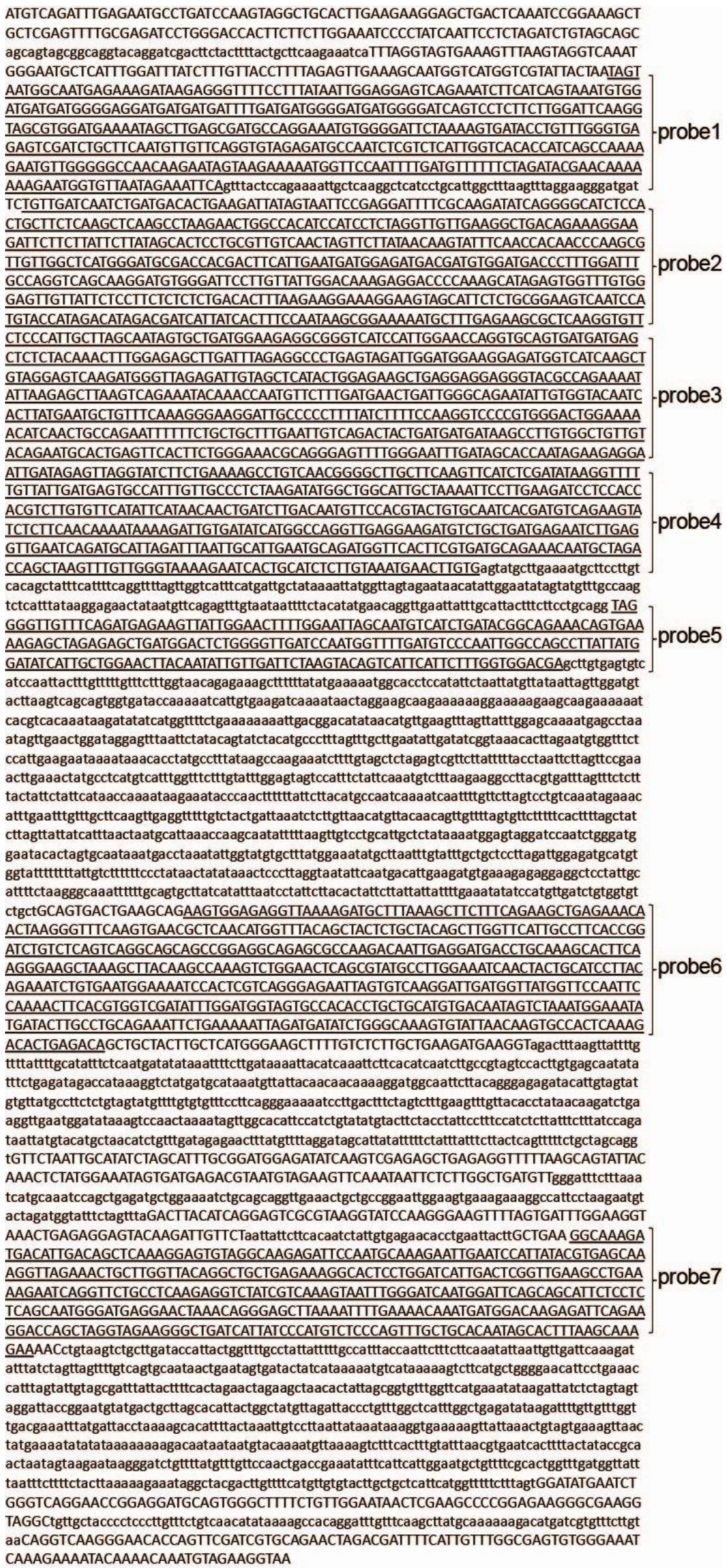

Fig. S3. Genomic sequence of *ψGaSTI.*

The target sequences in the Northern blots are underlined. The theoretical coding sequence is uppercased.

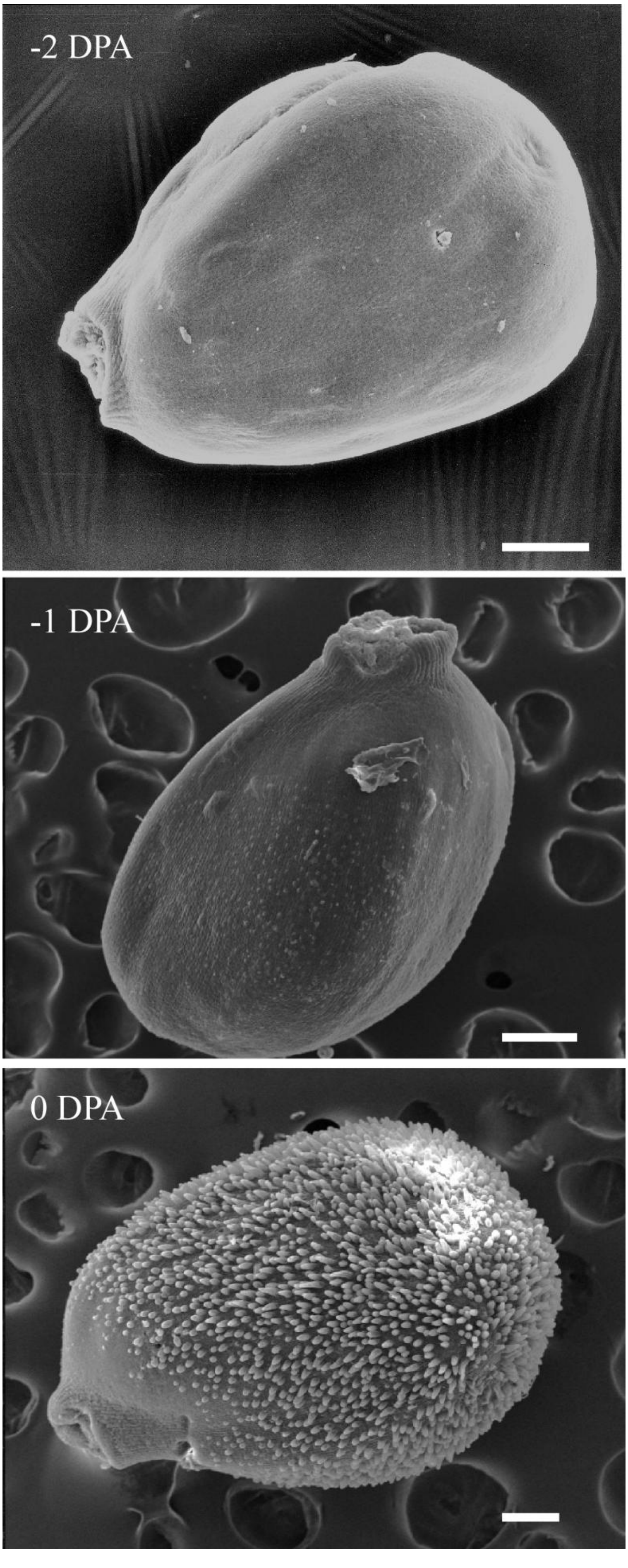

Fig. S4. Histological observation of DPL971 seeds at different developmental stages. Bars = 200 μm.

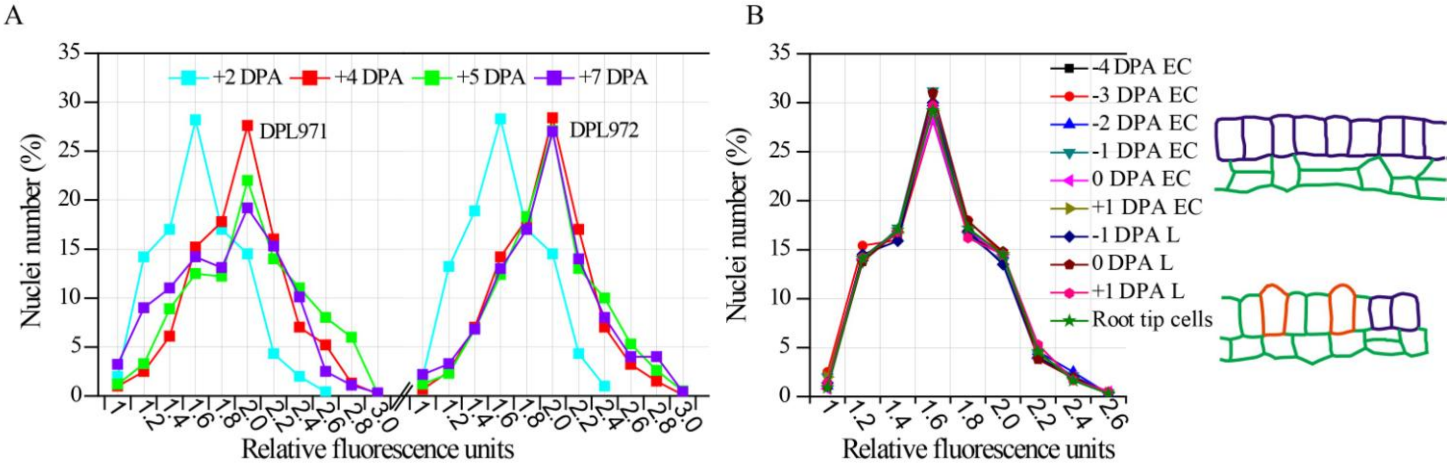

Fig. S5. Fiber nuclear DNA content at different developmental stages.

(A) Histograms showing the fluorescence of isolated nuclei from fiber cells at 2, 4, 5, and 7 DPA labeled with Hoechst 33258, expressed as a percentage of the total number of nuclei measured. The Relative fluorescence units (RFU) of DPL971 at 2, 4, 5, and 7 DPA was 1.62 ±0.06, 1.95 ±0.07, 1.90 ±0.06, and 1.82 ±0.06, respectively. The RFU of DPL972 at 2, 4, 5, and 7 DPA was 1.60 ±0.06, 1.92 ±0.07, 1.95 ± 0.06, and 1.93 ±0.09, respectively. The number of nuclei measured were all more than 400, and came from at least 7 ovules of different bolls.

(B) Histograms showing the fluorescence of isolated nuclei from different epidermal cells types before 2 DPA. EC, epidermic (unswelled) cells; L, lint cells

Red cells in the right panel are schematic diagrams of fiber initial cells and blue demonstrated non-fiber epidermal pavement cells. The two type cells were respectively collected by laser-capture microdissection. None of the pairwise comparisons among these samples is significant ($P > 0.05$, Kruskal-Wallis one-way ANOVA and Dunn′s test), approximately 1.63.

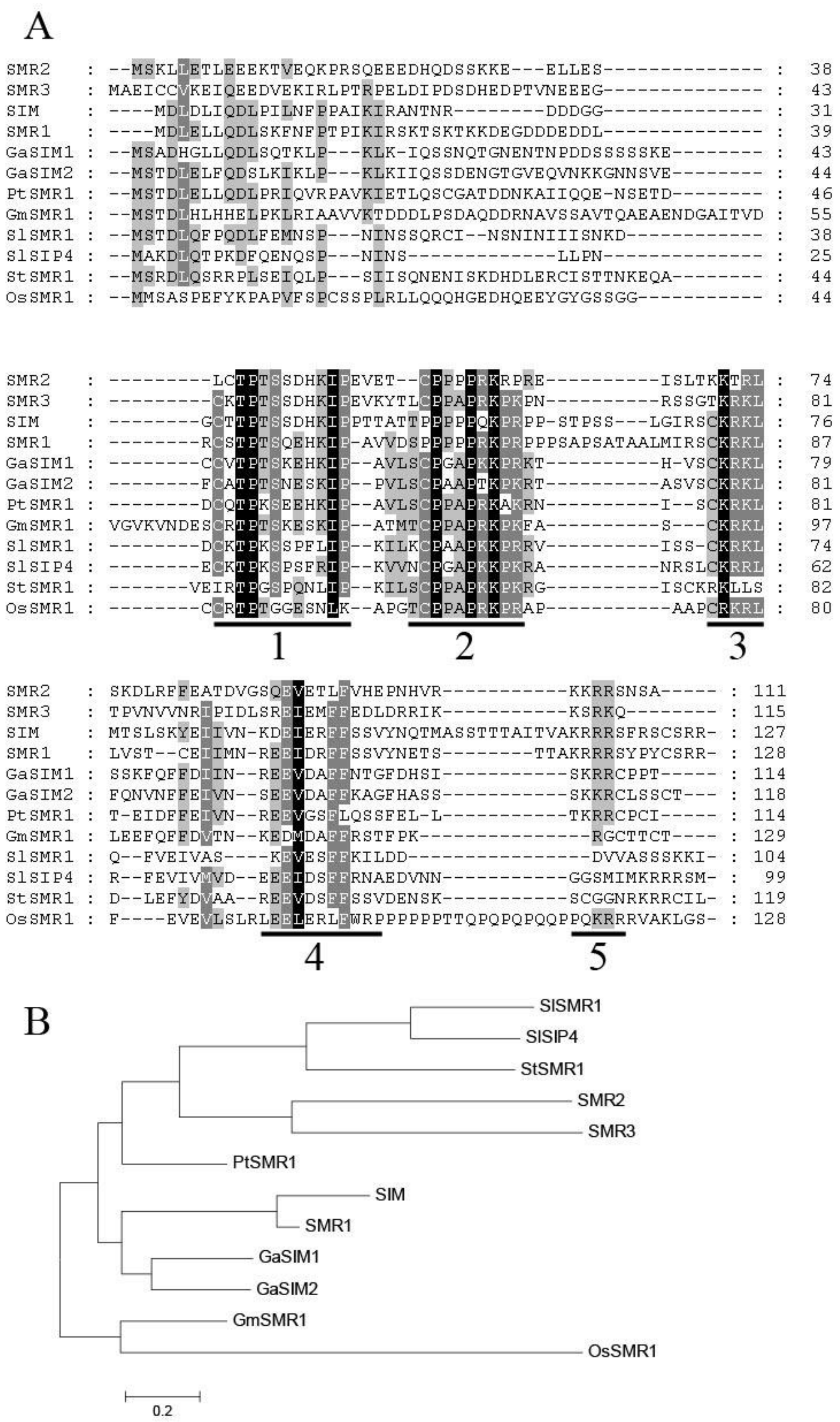

Fig. S6. Alignment and evolutionary tree of GaSIM related plant proteins.

(A) Alignment of conceptual translation of GaSIMs reading frame and related plant proteins. The regions numbered 1 to 5 denote conserved domains (Churchman *et al.*, 2006). SIM, CAB85553; SMR1, BAC42937; SMR2, AAF18255; SMR3, CAB85979; *Solanum lycopersicum* (Sl) SMR1, AI780963; SlSIP4, AAG43410; *S. tuberosum* (St) SMR1, BM110486; *Oryza. sativa* (Os) SMR1, AAK20052; *Populus tremula* (Pt) SMR1, BU815024; *Glycine max* (Gm) SMR1, AW704877. All of these accession numbers are from the National Center for Biotechnology Information database.

(B) A maximum likelihood tree was constructed in MEGA 5.05.

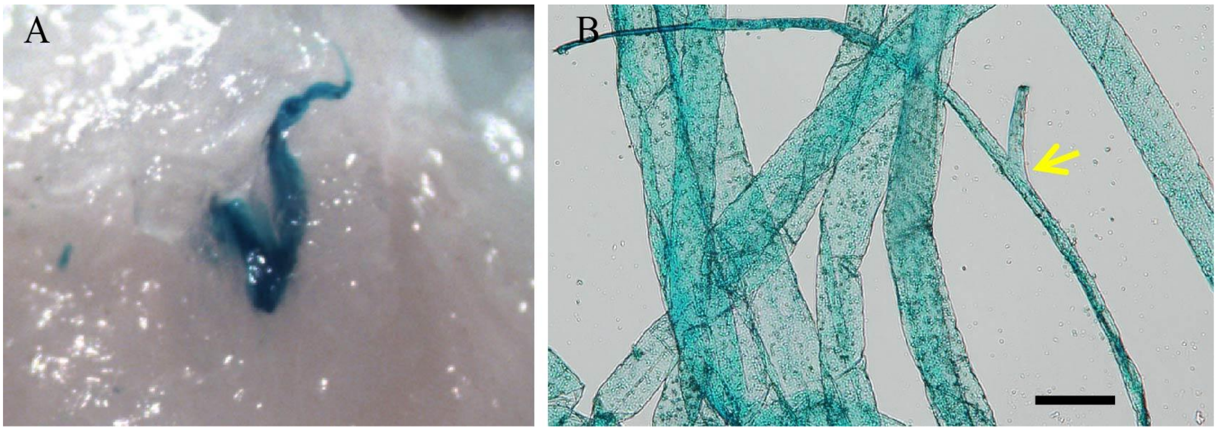

Fig. S7. Co-expression of *GaBLT, GaSIM, STI* can induce fiber-branching phenotype.

Ovules were collected at 2 DPA and bombard with a construct to target express *GaBLT*, *GaSIM1*, *Arabidopsis STI*, and *GUS* in fiber cell, followed by *in vitro* culture for 5 day. Fibers showing transgenic expression, indicated by GUS blue. A showing a tuft GUS stained fibers. B, a higher magnification of the fiber-tuft in A.

Yellow arrow shows branching site. Bar = 30 µm.

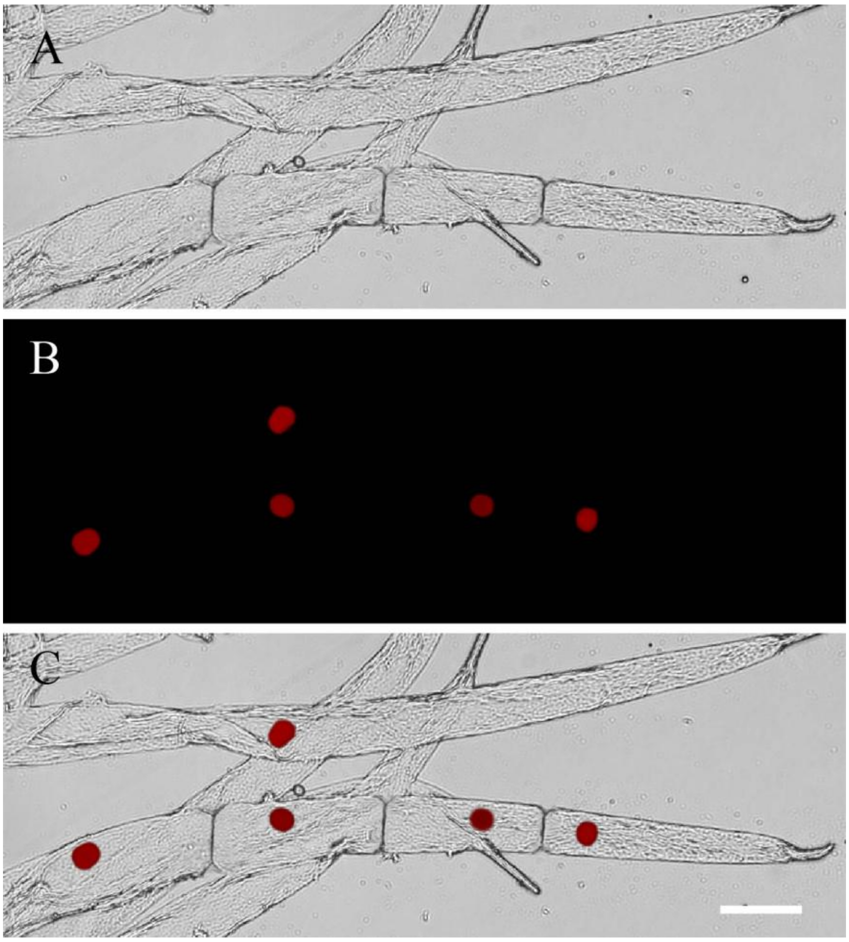

Fig. S8. Fibers with mulitcelled and unicelled phenotype.

(A) Light micrograph. (B) Propidium Iodide (PI) image. (C) Overlay.

5 DPA Ovules were removed from the boll and fixed in methanol:glacial acetic acid (3:1). Then rinsed with 1×PBS, followed by PI(50 μg/ml) staining (10 min). PI images were acquired using a CCD camera (Olympus DP72) attached to anOlympus microscope (BX53). The equipment was operated with cellSens standard software (ISO 200; exposure time 380 ms). Bar = 30 μm.

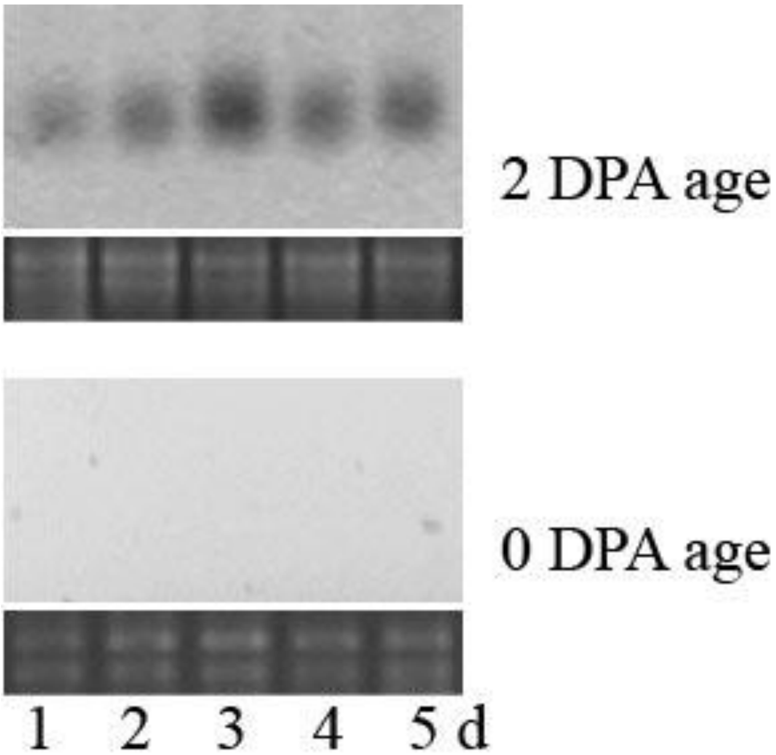

Fig. S9. Northern blots with antisense-*GaBLT* probe showing *GhBLT* mRNA levels in cultured fibers. The ovules of *G. hirsutum* cultivar MD51 ne were put into culture at 2 DPA (2 DPA age) or 0 DPA (0 DPA age) for 1 to 5 days, and then recover fiber RNA for detection.
